# Identification of Putative Molecular Markers Associated with Root Traits in *Coffea canephora* Pierre ex Froehner

**DOI:** 10.1155/2015/532386

**Published:** 2015-03-03

**Authors:** Devaraja Achar, Mallikarjuana G. Awati, M. Udayakumar, T. G. Prasad

**Affiliations:** ^1^Department of Crop Physiology, University of Agriculture Sciences, Gandhi Krishi Vignana Ken-dra, Bangalore, Karnataka 560 065, India; ^2^Division of Plant Physiology, Central Coffee Research Institute, Balehonnur, Chikmagalur District, Karnataka 577 112, India

## Abstract

*Coffea canephora* exhibit poor root system and are very sensitive to drought stress that affects growth and production. Deeper root system has been largely empirical as better avoidance to soil water limitation in drought condition. The present study aimed to identify molecular markers linked to high root types in *Coffea canephora* using molecular markers. Contrasting parents, L1 valley with low root and S.3334 with high root type, were crossed, and 134 F1 individuals were phenotyped for root and associated physiological traits (29 traits) and genotyped with 41 of the 320 RAPD and 9 of the 55 SSR polymorphic primers. Single marker analysis was deployed for detecting the association of markers linked to root associated traits by SAS software. There were 13 putative RAPD markers associated with root traits such as root length, secondary roots, root dry weight, and root to shoot ratio, in which root length associated marker OPS1_850_ showed high phenotypic variance of 6.86%. Two microsatellite markers linked to root length (CPCM13_400_) and root to shoot ratio (CM211_300_). Besides, 25 markers were associated with more than one trait and few of the markers were associated with positively related physiological traits and can be used in marker assisted trait selection.

## 1. Introduction

The world's primary source of caffeine is the coffee “bean,” which is the seed of the coffee plant, from which coffee is brewed and therefore it is one of the most important commodities in the international agricultural trade, representing a significant source of income to several coffee growing countries. The genus* Coffea* belongs to Rubiaceae family which has 500 genera and over 6,000 species, in which* Coffea arabica* (2*n* = 44) and* Coffea canephora* (2*n* = 22) are the two commercially cultivated species. Currently the production of arabica and robusta coffee accounts for 65% and 35%, respectively; however arabica coffee typically contains half the caffeine of the robusta variety [1, 2]. The world coffee production in 2012/2013 was 155,140 (in 1,000 60 kilogram bags), but then in 2013/2014 and 2014/2015 there is a decline in coffee production and it is predicted to be decreased. This decreased coffee production is predominantly associated with variable climatic conditions, particularly limited water stress (drought), where water shortages are responsible for the greatest crop losses around the world [3, 4]. In several coffee growing countries drought is considered to be the major environmental stress affecting coffee production, in particular* Coffea robusta*. Because the robusta coffee is shallow rooted, it has largely been cultivated in water constraint, experiencing the stress at various crop phenology, which impacts crop growth and development above ground [5–7].

Indeed drought-adapted plants are often characterized by deep and vigorous root systems, since root associated traits play a crucial role in maintaining canopy hydraulic conductance with high carbon assimilation in drought [8, 9]. Breeding coffee plants for root traits to enhance the productivity under water stress is very much required, but little progress has been achieved due to quantitative nature and poor knowledge of the genetic control of drought tolerance. Furthermore, the quantitative nature of root traits could be either constitutive or adaptive, but difficult to phenotype. Therefore, it is not surprising that a majority of genetic research has focused on aboveground traits while the “hidden half” of the plant is much less represented in recent research [10]. With the conventional breeding methods, introgression of complex/quantitative traits is unfeasible. Molecular marker technology and genomics serve as a tool for selecting such complex traits and allow breeders to track genetic loci controlling drought resistance traits, without measuring the phenotype, thus reducing the need for extensive field testing over space and time [11–13]. With the functional genomics it was shown that the increase in the amount of* RBCS1* (Rubisco small subunit) protein could contribute to the antioxidative function of photorespiration in water-stressed* Coffea canephora* plants [14]. Furthermore it was also shown that drought acclimation in* Coffee canephora* clones probably involving the abscisic signalling pathway and nitric oxide are major molecular determinants that might explain the better efficiency in controlling stomata closure and transpiration displayed by drought-tolerant clones of* C. canephora* [15].

With an advent of Next Generation Sequencing (NGS) technologies,* Coffea canephora* is the first coffee species fully sequenced [16]. The developed crop-specific hub, the Coffee Genome Hub (CGH) (http://coffee-genome.org/), can be exploited to identify the genes/SNP markers conditioning for drought and root associated secondary traits by resequencing intra- and interspecific genetic resources of robust coffee species [17]. Therefore, it is very important to identify the genetic loci conditioning root associated traits for breeding better drought tolerance clones. With this background, our study aims at (1) developing a mapping population with contrasting parental lines for root traits and phenotyping, (2) genotyping the mapping population with RAPD and SSR markers, and (3) detecting the association of molecular markers with complex physiological and morphological traits.

## 2. Materials and Methods

### 2.1. Plant Materials

To identify the markers linked to root and associated physiological traits, F1 mapping population was developed by crossing low root type L1 valley as female parent with high root type S.3334 as male parent. After successful pollination, matured 300 coffee fruits were sown in nursery and after 48 days and 135 healthy seedlings were transplanted to carbonized rubber containers (35 kg capacity) for better establishment. This experiment was conducted at Central Coffee Research Institute, Chikmagalur District, Karnataka State, India. Institute is situated at 13°22′′ north Latitude and 75°28′′ east Longitude at an elevation of 824 to 884 meters above mean sea level.

### 2.2. Phenotypic Analysis

The moisture regime of 70% field capacity (FC) was imposed for 180 days after six months of coffee seedlings established in carbonized rubber containers. Gravimetric approach was followed to maintain 70% FC, where potted plants were daily weighed to add water which was evapotranspired [18].

### 2.3. Observations Recorded during Treatment Period

During the treatment period (180 days), cumulative water added, evaporation, evapotranspiration were recorded. After the treatment period, root traits such as root length (cm), number of secondary roots, root dry weight (g/plant), shoot dry weight (g/plant), and root to shoot ratio were recorded. Besides, morphological, gravimetric, and gas exchange parameters were also recoded [19].

### 2.4. Genomic DNA Extraction

Coffee leaves were frozen in liquid nitrogen and stored at −80°C. DNA was extracted from frozen leaves using cetyltrimethyl ammonium bromide (CTAB) method [20]. For the CTAB technique, 900 *μ*L of CTAB extraction buffer was added to lyophilized leaf tissue in 2 mL Eppendorf tubes and then lightly vortexed. The tubes were placed in hot water bath (65°C) for 45 min and mixed with 400 *μ*L of chloroform : isoamylalcohol (24 : 1) and centrifuged for 15 min. The aqueous layer was collected, and 800 *μ*L of isopropanol was added to precipitate the nucleic acids. Nucleic acid pellets were washed with 400 *μ*L of 70% ethanol, dried, and resuspended in 100 *μ*L of Tris-EDTA buffer (10 mM Tris with pH 7.5 and 0.5 mM EDTA).

### 2.5. Polymerase Chain Reaction (PCR)

RAPD (Random Amplified Polymorphic DNA, Operon Technologies) primers were used to genotype mapping population. Polymerase chain reaction was carried out in 15 *μ*L reaction containing 1x buffer, 2 mM dNTPs, 2.5 mM MgCl_2_, 5 *μ*M primer, and 1 U* Taq* DNA polymerase (NEB). Amplification was performed with the following thermal cycle profile: 94°C/4 min hot start denaturation, followed by 35 cycles of 94°C for 1 min, primer annealing at 38°C for 1 min, extension at 72°C for 2 min, and a final extension at 72°C for 8 min. The PCR was performed using Eppendorf thermocycler (Eppendorf, Hamburg, Germany). The PCR products were run on 1.5% agarose gel at 90 volts for 1 h 30 min and amplified fragments were documented using Hero Lab Gel Documentation system (Inkarp).

SSR (simple sequence repeats) primers for polymerase chain reaction were synthesized based on the information available in coffee genome database and also from in-house developed from S.3334 coffee accession.

PCR amplification was carried out with 15 *μ*L reaction mixture having 50 ng DNA, 1x PCR buffer, 100 *μ*M dNTPs, 250 *μ*M primers, and 1 unit Taq polymerase enzyme (NEB). Amplification was performed with the following thermal cycle profile: 95°C for 5 min, followed by 35 cycles of polymerization reaction, each consisting of denaturation at 94°C for 15 s, annealing at 60°C for 45 s, and an extension step at 72°C for 1 min. A final extension step was run for 5 min at 72°C. The PCR was performed using Eppendorf thermocycler (Eppendorf, Hamburg, Germany). The PCR products were run on 6% polyacrylamide denaturing gels. Amplified fragments were detected using a silver-staining procedure (Promega, Madison, WI, USA).

### 2.6. Study of Parental Polymorphism

The contrasting parents (L1 valley × S.3334) for root traits were screened with 320 RAPD (series from OPK1 to OPZ20) and 55 SSR markers. The polymorphic RAPD bands were visually scored for the presence (1) or absence (0) and in SSR analysis the segregating band from the female parent was scored as 3, male parent as 1, and heterozygous bands were scored as 2 in all individuals of F1 mapping population. The binary data was used for further statistical analysis. Based on the segregation of RAPD markers in the mapping population, the putative genotypic interpretation of the parents for the marker locus was made and the Chi-square test was performed (http://www.physics.csbsju.edu/stats/chi-square_form.html).

### 2.7. Association of Identified Polymorphic Markers with Physiological Traits

Single point analysis [21, 22] for detecting the association of molecular markers with complex physiological and morphological traits was done using SAS software. To find the amount of variability explained by these markers, regression (*R* ^2^) values were worked out by one-way analysis of variance (ANOVA), by general linear model (GLM) procedure. In this analysis, different traits were treated as dependent variable and the various molecular markers as independent variables. A total of 30 different physiological and morphological traits were used to associate with the 85 polymorphic molecular markers [23].

## 3. Results

### 3.1. Phenotyping Root Length, Secondary Roots, Root Dry Weight, and Physiological Traits

**Figure 1:**
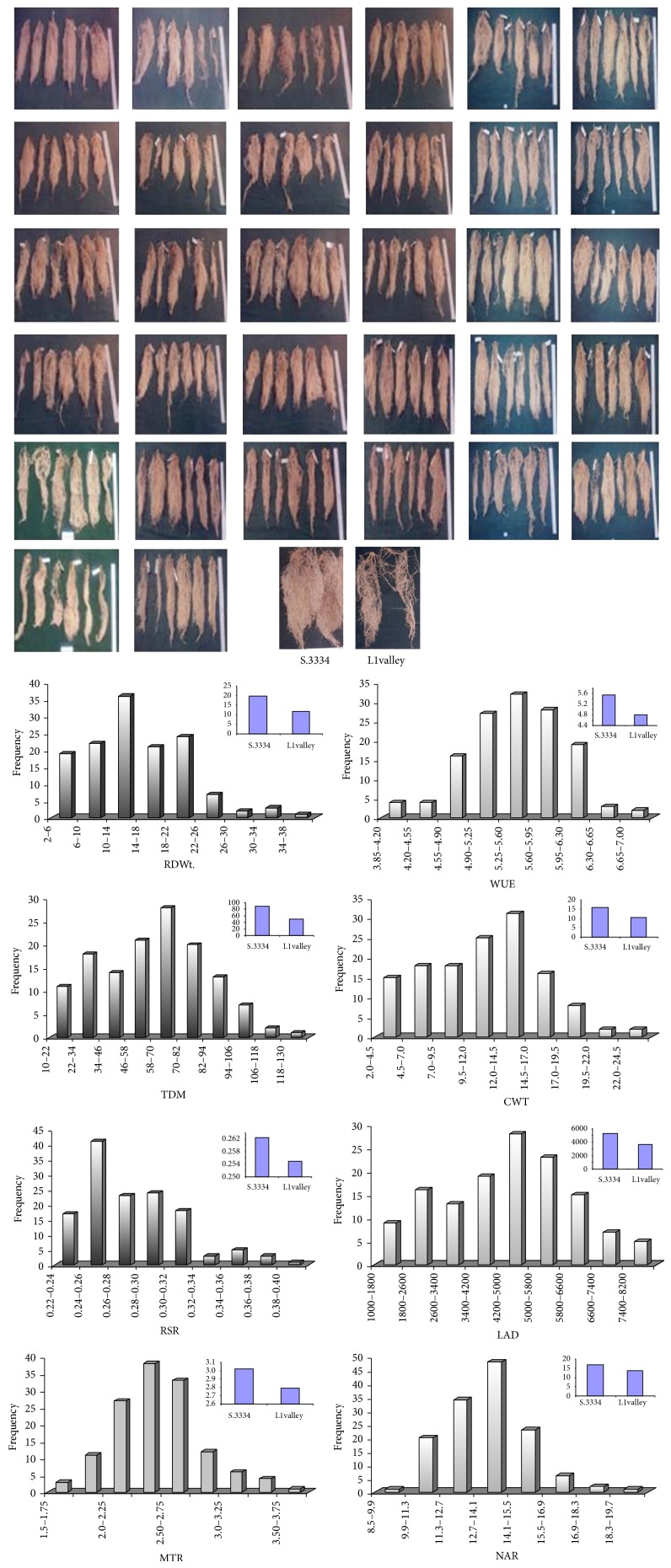
Genetic variability of root system and quantitative traits of mapping population of L1 valley × S.334.

At the end of experiment period about 180 days water stress, pots were completely saturated with water and then, in next day, all plants were carefully detached with water force to avoid root loss. The data on root length, secondary roots, and root biomass data of parental lines (Table 1) and F1 mapping population were recorded (Table 2). There was significant genetic variability observed in root length, ranging from 41.5 cm to 83.0 cm with a mean of 57.08 cm. Similar trend was followed in dry weight, ranging from 2.57 g to 34.69 g with a mean of 13.81 g/plant, and root to shoot ratio varies from 2.57 to 4.41 with mean ratio of 3.77. It was observed that F1s root traits were distributed between the values of the respective parental lines without much considerable transgressive segregation (Figure 1). The quantitative nature of these traits showed continuous variation, confirming the metric nature of the traits.

Morphological traits such as plant height, number of nodes and leaves, stem girth, internodal length, leaf area, shoot dry weight, and total dry matter were recorded. Nonetheless, all these traits showed significant and continuous variation (Table 2). Besides, genetic variability in the mapping population in net photosynthesis (Pn), stomatal conductance (g_s_), transpiration rate (*E*), intrinsic WUE (Pn/g_s_), and mesophyll efficiency (Ci/g_s_) was observed at single leaf level.

Significant variations were observed in water use efficiency (WUE) and associated physiological traits like cumulative water transpired (CWT), net assimilation rate (NAR), mean transpiration rate (MTR), leaf area duration (LAD), and Δ^13^C in the population. The WUE ranges from 3.86 to 6.84 g/kg with an average of 5.40 g/kg and considerable transgressive segregation was observed. The variation in CWT was observed between 2.50 and 23.50 kg/plant. The average transpiration rate was 10.81 L/plant in the population. The NAR and MTR also varied with mean values of 13.07 mg/dm^2^ and 2.44 mL/dm^2^ of leaf area, respectively. Similarly, the functional leaf area (LAD) varied between 1065.89 and 7992.77 dm^2^/day with mean leaf area duration of 4376.57 dm^2^/day. Δ^13^C also varied between 19.75 and 26.53‰ with a mean of 21.80‰ (Table 2). The variation in WUE, CWT, Δ^13^C, and so forth, showed that traits are metric nature and mapping population could be utilized for the identification of marker linked to quantitative traits.

### 3.2. Association of Root with Other Physiological Traits

**Figure 2:**
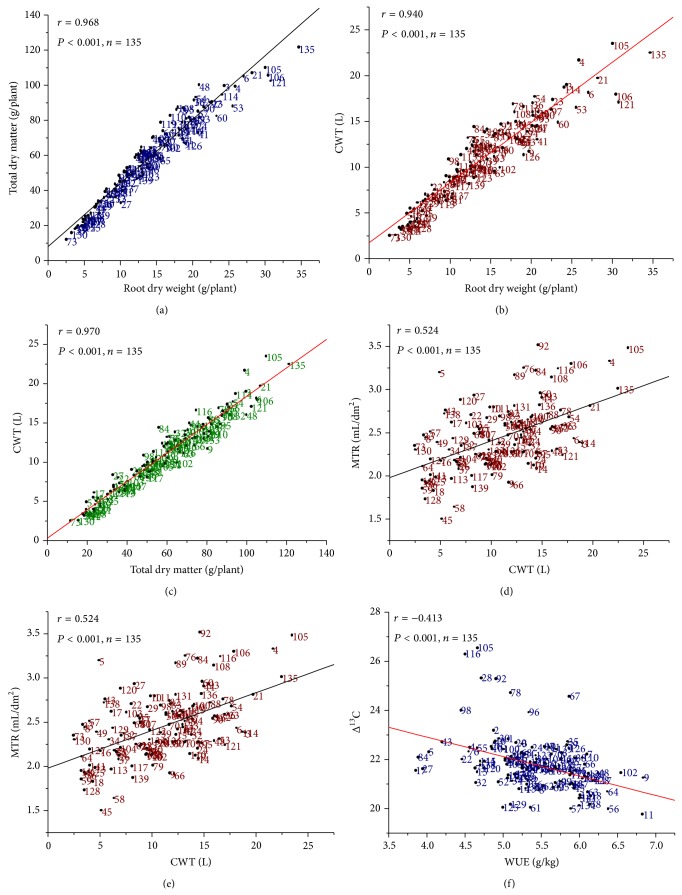
Relationship between the RDW/TDM, RDW/CWT, TDM/CWT, CWT/MTR, CWT/MTR, and WUE/Δ^13^C in the mapping population of* C. canephora* (L1 valley × S.334).

The plants with the higher root biomass showed higher total biomass and correlation between these two traits is highly significant (*r* = 0.968) (Figure 2). It signifies the importance of root system in determining the total dry matter by absorbing the water from sub-soil layers from its better root system compared to parental lines. Subsequently strong positive significant correlations were noticed between root weight and total transpiration (*r* = 0.940), CWT and MTR (*r* = 0.524), and total dry matter and CWT (*r* = 0.970) (Figure 2), suggesting that maintaining canopy hydraulic conductance with high carbon assimilation is one of the drought tolerance mechanisms.

### 3.3. Relationship between WUE with Other Physiological Traits

Significant inverse relationship between WUE and Δ^13^C (*r* = −0.413) was observed (Figure 2). It suggests that Δ^13^C could be a strong surrogate measure for WUE even in mapping population. The relationship between CWT and WUE was poor, suggesting stomatal control of WUE in mapping population and, similarly, between root weight and WUE. However, significant negative relationship between MTR and WUE was observed (*r* = −0.552) in mapping population (data not shown). This signifies the genetic nature of the coffee plants for its heritable conductance type. It suggests that stomatal factors regulate the WUE in these plants rather than photosynthetic efficiency (mesophyll factors).

### 3.4. Identification of Polymorphic RAPD and Microsatellite Primers in Parental Lines

The contrasting parental lines L1 valley (low root type) and S.3334 (high root type) were genotyped with 320 RAPD primers, of which 41 polymorphic primers generated 70 polymorphic loci (Table 3). Since RAPD is dominant marker, the PCR amplified DNA fragments were scored as dominant (AA/Aa), whereas absence of bands was scored as recessive (aa). We have analyzed our data within the framework of these assumptions.

Among them more than 85% of the primers produced single polymorphic locus and 15% of the primers yielded more than three polymorphic loci.

All the RAPD markers in the F1 generation of* C. canephora* segregated in ratios that were not consistent with Mendelian inheritance. The Chi-square tests were performed for each marker to determine segregation distortion from the expected allele frequency ratio of 1 : 1. At a significance level of *P* = 0.05, about 62% of the marker loci were in agreement with the expected ratios and 38% of the marker loci were not following the Mendelian inheritance (Table 4). It is expected that RAPD bands of maternal DNA origin would show non-Mendelian inheritance.

Besides RAPD marker system, 55 SSR markers were also used for genotyping; however, only nine primers showed polymorphism (Table 5), but did not follow the Mendelian segregation. The low level of DNA polymorphism between the two parental accessions, coupled with the large number of common bands, implies parental lines could be less diverse.

### 3.5. Single Marker Analysis for Root and Associated Physiological Traits

Complex physiological traits have been described by a small number of major QTL [24], but it is intricate to find useful QTL for a particular trait, as their individual contribution is smaller. Therefore under such condition Single Marker Analysis (SMA) is generally a good choice when the goal is simply a detection of marker locus linked to a trait. However, estimation of its position and its effects requires further complex analysis such as marker regression [25] or interval analysis [26].

In our study Single Marker ANOVA is used to identify markers showing significant association with 29 morphophysiological traits (SAS software with *P* ≤ 0.05).

In the single marker analysis, simple regression model was examined to study the association between marker loci (independent) and trait score (dependent variable) and also computed the percent phenotypic variance explained by each marker. Seventy polymorphic marker loci were employed for simple regression analysis; however, it was discovered that 37 RAPD and 5 SSR markers explained their association with morphophysiological traits.

### 3.6. Markers Linked to Root Traits

The single marker analysis revealed four markers such as OPS1_850_, OPK11_780_, OPY20_1200_, OPZ10_1350_, and CM13_400_ were significantly associated with root length (*P* = 0.05) and explained phenotypic variance of 4.41%, 2.95%, 3.56%, 5.12%, and 5.12%, respectively. A number of secondary roots linked to three markers such as OPO16_450_ (4.40%), OPK11_1000_ (5.51%), and OPV14_550_ (5.82%) were significantly (*P* = 0.05) associated. Similarly six markers such as OPK11_1000_ (3.26%), OPP9_1030_ (3.5%), OPM11_600_ (3.75%), OPL19_900_ (5.61%), OPL19_600_ (4.11%), OPR4_600_ (3.30%), and CM211_300_ (*R* ^2^ = 4.96%) revealed significant (*P* = 0.05) association with the root to shoot ratio (Table 6). This implies the root associated traits are polygenic controlling many genes. However, root dry weight is associated with only one maker OPL1_1400_ (4.86%).

### 3.7. Markers Linked to Morphophysiological Traits

Besides root traits, polygenic inheritance traits such as morphological, gravimetric, and gas exchange at single leaf level traits were also deployed for marker trait association. Single marker analysis revealed more than three markers linked to each trait and 25 markers are colocalized with more than one trait and 17 markers are associated with single trait (Table 7). The marker OPP5_1800_ is coassociated with shoot dry weight and leaf area duration (LAD), and these two traits are positively correlated (*R* ^2^ = 0.93). Four markers (OPL1_1450_, OPL19_1650_, OPM17_1400_, and OPK20_350_) linked to NAR and two markers OPR4_600_ and OPL12_2000_ are significantly (*P* = 0.01) associated with cumulative water transpired (CWT).

The ^13^ΔC is the surrogate method to quantify the water use efficiency and it was demonstrated that there is inverse relationship between these two traits. Of the six associated markers, OPZ10_1350_ was found to be associated with ^13^ΔC and WUE with phenotypic variance of 4.12% and 3.02%, respectively. This marker locus could be negative additive effect since traits are negatively related. The mesophyll efficiency (Ci/g_s_), intrinsic WUE (A/g_s_), and photosynthetic rate (Pn) are, respectively, associated with CM13_400_ (4.83%), CM171_210_ (3.45%), and CM171_190_ (3.72%) (Table 7). Whereas positively related traits are associated with the same marker such as OPP5_1800_ (LAD and shoot dry weight), OPZ10_1030_ (MTR and g_s_), and OPK20_350_ (Ci and NAR) alleles contribution from S.3334. The markers linked to other gas exchange parameters were given in Table 7, which are contributed from S.3334.

## 4. Discussion

We developed a F1 mapping population by crossing contrasting root traits S.3334 high root type as male and L1 valley low root type as female to identify makers linked to root traits and water use efficient types. However, in annual crops, F_2_, recombinant inbred lines (RILs), near isogenic lines (NILs), and doubled haploids (DH) mapping population will be used to associate markers with the trait of our interest. However, in perennials the germplasm lines and the cultivated accessions are often highly heterozygous; consequently F_1_ population developed from heterozygous parents was used in this study and showed considerable phenotypic and genetic variations.

One of the objectives of studying the mapping population was to score the variability in roots and other physiological traits under drought and thus mapping population was gown up in 70% FC of water stress to study the heritability of root traits. Indeed root traits are highly complex genetic mechanism, controlled by multi genes and highly heritable in water non limiting environment [27]. The results demonstrated significant variability was observed in root dry weight (RDW), total dry matter (TDM), root to shoot ratio (RSR), cumulative water transpired (CWT) with considerable transgressive segregation (Figure 1), and quantitative inheritance of drought tolerance [28]. However, significant positive correlation between RDW/TDM, RDW/CW, and TDM/CWT indicates that under drought increased root growth maintains the shoot hydraulic conductance as adoptive strategy [29, 30]. The decreased mean transpiration (MTR) and increased water use efficiency (WUE) could be due to decreased stomatal aperture through chemical signals such as ABA [31]. However, some of the clones showed that increased MTR with increased CWT would favor the stomatal conductance and net photosynthesis. Such clones are better for breeding drought tolerance. Inverse relationship between Δ^13^C and WUE in robusta coffee accessions was observed (Figure 2), thus measuring Δ^13^C which can be a surrogate method to identify best WUE types clones in breeding [19, 32]. Breeding for such quantitative traits is highly complex cumbersome and thus by determining the allele of a DNA marker, plants that possess particular genes or quantitative trait loci (QTLs) are better than their phenotype. With this background effort was made to identify the markers linked to root and associated physiological traits in* Coffea canephora*.

Despite the efforts, no successful linkage map could be obtained, partly due to the inadequate number of polymorphic markers generated from this study. The output of the association analysis indicates that, out of 29 traits, only 24 traits were linked to 85 markers. Even markers linked to traits explaining the phenotypic variation were not more than 10%. In the present study, phenotypic variation explanation for all the traits ranges from 2.61 to 9.86%. This suggests that the successful application of molecular markers in trait mapping greatly requires more polymorphic markers, while successful mapping attempts could still be carried out in the absence of a linkage map as done in chickpea [33]. Although construction of linkage map was done in* C. arabica* [34] and* C. canephora* [35] predominantly using AFLP and RAPD markers, association with the traits was not attempted. Similarly the first molecular linkage map generated using pseudo testcross strategy for* Coffea canephora* (CxR × Kagnalla) has the largest number of mapped SSRs (71 genomic, including nine EST-SSRs) [36], but trait association was not correlated.

Most of the markers explained phenotypic variance between 3 and 4%; however, only two markers, that is, OPZ11_1500_ and OPV14_500_, were significantly (*P* < 0.01) linked to photosynthetic rate (Pn), accounting the variation of about 9.23% and 9.30%, respectively (Table 7). The root traits such as root length, number of secondary roots, root dry weight, and root: shoot ratio, revealed significant association (*P* < 0.05) with markers and the variability ranges from 2.95 to 6.86% (Table 6). This suggests the trait variability explanation is not sufficient to answer the probabilities of occurrence of these markers due to limited polymorphic markers.

In the present study, codominant SSR markers were also used for the marker-trait association study. The SSR markers identified herein are significantly associated with morphological and physiological traits, but these markers were explaining the trait in the range between 2.83% and 7.19%. This study has provided more detailed information of root and associated physiological traits relationships under drought and identification of markers linked to such traits with the limited polymorphic markers by employing Single Marker Analysis (SMA). QTL maps could be used to for long-term, drought breeding. Further studies are needed to confirm the estimation of QTL positions and effects and to validate markers prior to routine marker assisted selection for drought tolerance in coffee.

## Figures and Tables

**Table 1:** Genotypic variation of parental accessions S.3334 (high root type) and L1 valley (low root type).

		S.3334	L1 valley
1	Root length	45	30
2	Secondary roots	40	15
3	Root dry weight	31.81	4.95
4	Root to shoot ratio	0.41	0.36
5	Total dry matter	106.77	15.49
6	Cumulative water transpired	28.86	31.15
7	water use efficiency	3.72	3.62
8	Net assimilation rate	13.75	9.09
9	Mean transpiration rate	3.72	2.23
10	Pn/g_s_	78.39	43.78
11	Pn/*E*	2.99	1.84
12	Ci/g_s_	3863.5	3399.4

**Table 2:** Range and mean values of the morphophysiological parameters in 134 F1 mapping population of *Coffea canephora* (L1 valley × S.3334).

	Serial number	Parameters	Range	Mean	STDEV
Root traits	1	Root length (cm^2^)	41.5–83.0	57.08	7.29
	2	Number of secondary roots	2–60	26.9	12.61
	3	Root dry weight (g/plant)	2.57–34.69	13.77	6.6
	4	Root : shoot ratio	0.23–0.39	0.27	0.03

Morphological traits	5	Plant height (cm^2^)	13.5–105.3	72.6	15.69
	6	Number of nodes	11.0–42.0	24.08	6.88
	7	Number of leaves	2–70	34.02	13.42
	8	Stem girth (mm)	5.88–13.65	10.28	1.79
	9	Internodal length (cm)	1.9–12.5	8.54	2
	10	Specific leaf dry weight (mg/dm^2^)	1.41–2.64	1.8	0.22
	11	Final leaf area (cm^2^)	1136.92–8190.61	4491.19	1710.17
	12	Specific leaf area (cm)	378.99–710.97	562.44	65.34
	13	Total leaf dry weight (g/plant)	5.78–48.59	26.02	10.43
	14	Stem dry weight (g/plant)	4.81–52.62	24.33	10.4
	15	Shoot dry weight (g/plant)	10.59–96.64	50.35	20.63
	16	Total dry matter (g/plant)	11.6–121.43	57.93	24.77

Gravimetric traits	17	Leaf area duration (dm^2^ days)	1065.89–7992.77	4372.52	1658.04
	18	Net assimilation rate	8.9–19.38	13.07	1.71
	19	Cumulative water transpiration (lt/plant)	2.5–23.5	10.81	4.62
	20	Water use efficiency (g/kg)	3.86–6.84	5.4	0.58
	21	Mean transpiration rate (mL/dm^2^ days)	1.5–3.52	2.44	0.38
	22	Delta 13c (mill)	19.75–26.53	21.8	1.11

Gas exchange parameters	23	Photosynthesis (Pn). (mmol/m^2^/S)	5.45–27.27	16.9	3.91
	24	Transpiration rate (*E*) (mg/m^2^/s)	0.71–7.47	3.38	1.34
	25	Conductance (g_s_) (mmol/m^2^/s)	0.05–1	0.35	0.17
	26	Ci/g_s_	288.76–3484.25	859.3	405.1
	27	Ci	186.5–296.67	247.98	24.88
	28	A/g_s_	24.06–93.26	54.42	15.52
	29	A/*E*	2.63–14.07	5.52	1.76

**Table 3:** Polymorphic RAPD primers and their sequences used to study the segregation of markers in the mapping population.

Polymorphic bands	Primer name	Sequence	Primer name	Sequence	Primer name	Sequence	Primer name	Sequence
1	OPK18	CCTAGTCGAG	OPN6	GAGACGCACA	OPQ5	CCGCGTCTTG	OPV5	TCCGAGAGGG
	OPK19	CACAGGCGGA	OPO1	GGCACGTAAG	OPS1	CTACTGCGCT	OPW9	GTGACCGAGT
	OPK20	GTGTCGCGAG	OPO18	CTCGCTATCC	OPV11	CTCGACAGAG	OPY20	AGCCGTGGAA
	OPM5	GGGAACGTGT	OPO4	AAGTCCGCTC	OPV14	AGATCCCGCC	OPZ11	CTCAGTCGCA
	OPN19	GTCCGTACTG	OPP2	TCGGCACGCA	OPV2	AGTCACTCCC		

2	OPK10	GTGCSSCGTG	OPL18	ACCACCCACC	OPO16	TCGGCGGTTC	OPT7	GTCCATGCCA
	OPK11	AATGCCCCAG	OPM11	GTCCACTGTG	OPP5	CCCCGGTAAC	OPU1	ACGGACGTCA
	OPL1	GGCATGACCT	OPM17	TCAGTCCGGG	OPQ20	TCGCCCAGTC	OPW17	GTCCTGGGTT
	OPL12	GGGCGGTACT	OPO10	TCAGAGCGCC	OPR4	CCCGTAGCAC	OPZ3	CAGCACCGCA

3	OPL19	GAGTGGTGAC	OPM2	ACAACGCCTC	OPP11	AACGCGTCGG	OPP9	GTGGTCCGCA
	OPZ10	CCGACAAACC						

4	OPK17	CCCAGCTGTG						

**Table 4:** Chi-square tests (*χ* ^2^) for RAPD and SSR markers in the *Coffea canephora* mapping population.

Parents	F1 progenies	Marker system	Total polymorphic markers	Marker loci not following expected ratio	Marker loci following expected ratio	Number of significant markers		
						*P* = 0.95	*P* = 0.05	*P* = 0.001
L1 valley × S.3334	134	RAPD	70	28	42 (1 : 1)	32	3	7
		SSR	9	9	0 (3 : 1)	—	—	—

**Table 5:** Polymorphic microsatellite primers and their sequences used to study the segregation of markers in the mapping population.

Serial number	Primer	Sequence	Tm (°C)	Size range
1	CM11	F: GCTGCCAGAAAAATGTTGCAGTG	58	270–338
		R: CTGCCTCGTAAAAGCTTGCGTTG		

2	CM13	F: GCTATGCAGCTTGTTCGCAATCC	59	350–411
		R: CCAGCTATATCAGGAGCAGAACC		

3	CM180	F: CATGTGTAATACATTCAACAGTGA	60	300–350
		R: GCAATAGTGGTTGTCATCCTT		

4	CM32	F: AACTCTCCATTCCCGCATTC	60	180–200
		R: CTGGGTTTTCTGTGTTCTCG		

5	CM46	F: CAGCTAGTGTGAAGGGAAAC	55	300–350
		R: GTTATCATGGTCTTACACG		

6	CM20	F: CTTGTTTGAGTCTGTCGCTG	60	250–300
		R: TTTCCCTCCCAATGTCTGTA		

7	CM171	F: TTCCCCCATCTTTTTCTTTC	60	250–300
		R: TTGTATACGGCTCGTCAGGT		

8	CM166	F: AAGAGGTGCCTATCACCGTC	60	230–260
		R: CGAGGTATCAAAAAGCACCT		

9	CM211	F: TCATGCCAAATATGAGTGGA	60	300–350
		R: GAGATGGCAAAGGCTGTTC		

**Table 6:** RAPD markers linked to root traits in the mapping population of *C*. *canephora* (L1 valley × S.3334).

Trait	Marker	*R* ^2^ (%)	*P* < 0.05
Root length	OPY20_1200_	2.95	0.0449
	OPZ10_1350_	3.56	0.0286
	OPK11_780_	4.41	0.0178
	OPS1_850_	6.86	0.0001
	CPCM13_400_	5.12	0.0105

Number of secondary roots	OPO16_450_	4.4	0.0179
	OPK11_1000_	5.51	0.0059
	OPV14_550_	5.82	0.0042

Root dry weight	OPL1_1450_	4.76	0.0073

Root to shoot ratio	OPK11_1000_	3.26	0.038
	OPR4_600_	3.3	0.0367
	OPP9_1030_	3.5	0.0288
	OPM11_600_	3.75	0.0267
	OPL19_600_	4.11	0.0223
	OPL19_900_	5.61	0.0062
	CM211_300_	4.96	0.0105

**Table 7:** List of RAPD and SSR markers overlapped with more than one trait.

Markers	Traits	*R* ^2^ (%) (*P* < 0.05)	Markers	Traits	*R* ^2^% (*P* < 0.05)
OPK11_1000_	Internodal length	4.74	OPR4_600_ OPR4_750_	CWT	5.08
	Root to shoot ratio	3.26		RSR	3.30
	Number of secondary roots	5.51		Number of leaves	5.65
	Stem dry weight	3.44		g_s_	2.93
OPK18_1200_	Specific leaf area	4.70	OPV14_550_	Pn	9.30
	Plant height	4.51		Number of secondary Roots	5.82
OPK19_2000_	Photosynthetic rate (Pn)	5.55	OPY20_1200_	WUE	3.10
	WUE	4.43		Plant height	4.23
K20_350_	NAR	4.82	OPZ11_1500_	Number of leaves	3.99
	WUE	4.45		Root length	2.95
	Ci	4.32		Pn	9.23
OPL1_1400_	Plant height	5.77	OPZ10_1350_ OPZ10_1030_	MTR	8.21
	Internodal length	3.81		^ 13^ΔC	4.12
	NAR	4.27		Plant height	3.03
	Shoot dry weight	4.00		Root length	3.56
	Root dry weight	4.76		WUE	3.02
	Pn	2.61		Ci/g_s_	5.09
OPL12_2000_	Plant height	3.68		Pn	7.56
	CWT	4.74		MTR	4.38
OPL12_1650_	^ 13^ΔC	3.61	OPZ10_720_	g_s_	2.96
	pn	3.07		g_s_	3.26
OPL18_1000_	Number of nodes	7.78		Pn	3.14
	LAD	3.82		Internodal length	3.58
OPL19_900_	SLW	3.33	CPCM13_450_	Root length	5.12
	RSR	3.60		Ci/g_s_	4.83
OPM11_600_	A/g_s_	3.85	CM171_300_ CM166_260_	Stem girth	4.54
	RSR	4.11		A/g_s_	3.45
	MTR	2.94		No. of nodes	3.93
OPO10_1400_	Ci	3.13		SLA	7.19
	E	3.18		*E*	4.18
OPP5_1800_	LAD	3.82	CM211_300_	SLW	2.83
	Internodal length	5.82		RSR	4.96
	Shoot dry weight	2.71		*E*	4.23
OPP9_1030_	Ci/g_s_	6.12			
	SLW	3.33			
	RSR	3.50			
